# Changes in Salinity, Mangrove Community Ecology, and Organic Blue Carbon Stock in Response to Cyclones at Indian Sundarbans

**DOI:** 10.3390/life13071539

**Published:** 2023-07-11

**Authors:** Abhiroop Chowdhury, Aliya Naz, Seema B. Sharma, Rajarshi Dasgupta

**Affiliations:** 1 Jindal School of Environment and Sustainability, O.P. Jindal Global University, Sonipat 131001, India; 2 Jindal School of Liberal Arts and Humanities, O.P. Jindal Global University, Sonipat 131001, India; naazaliya6@gmail.com; 3 Department of Earth and Environmental Science, KSKV Kachchh University, Mundra Road, Bhuj 370001, India; 4 School of Public Policy, Indian Institute of Technology, New Delhi 110016, India

**Keywords:** biodiversity, climate change, coastal management, conservation, natural disaster, mangrove, blue carbon, nutrient pool, community ecology, mangrove ecology, geochemistry

## Abstract

Climate change-induced frequent cyclones are pumping saline seawater into the Sundarbans. Fani, Amphan, Bulbul, and Yaas were the major cyclones that hit the region during 2019–2021. This study represents the changes in the soil parameters, mangrove biodiversity and zonation due to the cyclone surges in the Indian Sundarbans between 2017 and 2021. Increasing tidal water salinity (parts per thousand) trends in both pre-monsoon (21 to 33) and post-monsoon (14 to 19) seasons have been observed between 2017 and 2021. A 46% reduction in the soil organic blue carbon pool is observed due to a 31% increase in soil salinity. Soil organic blue carbon has been calculated by both wet digestion and the elemental analyzer method, which are linearly correlated with each other. A reduction in the available nitrogen (30%) and available phosphorous (33%) in the mangrove soil has also been observed. Salinity-sensitive mangroves, such as *Xylocarpus granatum*,* Xylocarpus moluccensis*,* Rhizophora mucronata*,* Bruguiera gymnorrhiza*, and *Bruguiera cylindrica*, have seen local extinction in the sampled population. An increasing trend in relative density of salinity resilient, *Avicennia marina*,* Suaeda maritima*, *Aegiceras corniculatum* and a decreasing trend of true mangrove (*Ceriops decandra*) has been observed, in response to salinity rise in surface water as well as soil. As is evident from Hierarchical Cluster Analysis (HCA) and the Abundance/Frequency ratio (A/F), the mangrove zonation observed in response to tidal gradient has also changed, becoming more homogeneous with a dominance of *A. marina*. These findings indicate that cyclone, climate change-induced sea level rise can adversely impact Sustainable Development Goal 13 (climate action), by decreasing organic soil blue carbon sink and Sustainable Development Goal 14 (life below water), by local extinction of salinity sensitive mangroves.

## 1. Introduction

Mangroves are a plant group that possesses physiological or anatomical adaptations to survive in a hyper-saline tidal environment. These plants grow between the high tide level (HTL) and low tide level (LTL) along the tropical and subtropical coasts. Mangroves render several ecosystem services, such as protection against natural disasters and storm surges, blue carbon sequestration, a breeding ground for marine and estuarine biota, and consolidating soil against tidal currents [1]. Climate change is destabilizing both terrestrial and marine ecosystems worldwide. In some parts, it is increasing desertification along with the frequency of droughts, whereas on the tropical/subtropical coasts, it is increasing the frequency as well as the intensity of cyclones/typhoons/storm surges [2]. Caught between land and sea, mangrove ecosystems are the only natural barrier that humankind has against these coastal hazards. According to a study, 40 out of 100 people reside within 100 km of the coastal range [3]. Frequent storms/cyclones in these zones not only destroy this unique ecosystem but also affect the lives and livelihoods of the vulnerable population.

While Indonesia has the world’s largest share of mangrove cover (20.6%), the Sundarbans are among the most diverse and largest contiguous mangrove habitats in the world, and they are shared between India (40%) and Bangladesh (60%). The diurnal tide-washed riverine islands of the Indian Sundarbans were also designated as the Tiger Project in 1973, a UNESCO world heritage site in 1987, and a Biosphere Reserve in 1989, covering an area of about 9630 km^2^ [4,5]. It is home to around 100 tigers and 4.6 million people while being the most diverse (64 species, 42 genera, 29 families) contiguous mangrove patch.

Mangroves are sensitive to harsh environments, cyclone disturbance, salinity changes, and sea-level rise while protecting the mudflats from the vagaries of disasters [5]. Cyclones and storm surges result in salinity intrusion into the coastal region, which directly and indirectly alters the composition of the native mangrove community [6]. Any alterations in abiotic parameters can change the community ecology of an area [7]. These alterations may cause the extinction of natural salinity-sensitive mangroves [8]. Over the course of the five years (2017–2021), four major cyclones have devastated this region. The details are elucidated in Table 1. Cyclones push the saline seawater upward in the estuaries from the deep sea, as observed in past research [9,10]. Similarly, during cyclones and storm surges, saline water from the Bay of Bengal rushes into the inner and central parts of the Sundarbans, flooding the region with saline water [11]. These devastating cyclones and storm surges have increased the surface water salinity in the region [12,13,14,15].

Ecosystem function and services are dependent on the overall health of the ecosystem. With changing biodiversity due to salinity intrusion, regulating ecosystem services such as blue carbon sequestration may be impacted. Climate change has the potential to lower the carbon sequestration potential of the mangrove ecosystem along with changes in other biogeochemical cycles [16]. Changes in vegetation pattern can be estimated using standard phytosociological assessment methods [7,17]. Previous research on cyclone Aila (2009) has indicated the long-persisting increase in surface water salinity and its long-term impact on the changes in mangrove community composition, favoring the dominance of salinity-tolerant species in the central part of the Indian Sundarbans [5]. Sustainable Development Goals (SDG) outline the major areas for policy-level intervention to create a sustainable future. Changes in mangrove community ecology can impact the blue carbon sequestration, the major ecosystem service of mangroves (SDG 13: climate action) as well as impacting the diversity and distribution (SDG 14: life below water).

**Table 1:** Major cyclone that impacted in Indian Sundarbans from 2017 to 2021.

S.N.	Name of Cyclone	Wind Speed (km/h)	Date of Hit	Classification	Reference
1.	Fani	215	26 April 2019	Extremely Severe Cyclonic Storm	[11]
2.	Bulbul	110–120 gusting to 135	9 November 2019	Severe Cyclonic Storm	[18]
3.	Amphan	155–165 gusting up to 185	20 May 2020	Super Cyclonic Storm	[19]
4.	Yaas	maximum of 220	26 May 2021	Severe Cyclonic Storm	[15]

The hypothesis of this study is that a cyclone-induced salinity rise in surface water will decreases the blue carbon sequestration ecological function of mangroves, change the mangrove community ecology and zonation pattern in the mudflats. Thus, the aim of the present study is to trace the gradual changes in mangrove community ecology due to the changes in surface water salinity because of frequent cyclonic surges in the duration of four years from 2017 to 2021 and to access the alteration in ecosystem services (organic blue carbon sequestration) and ecosystem functions (nitrogen and phosphorus biogeochemical cycles).

## 2. Material and Methods

### 2.1. Study Site

**Figure 1:**
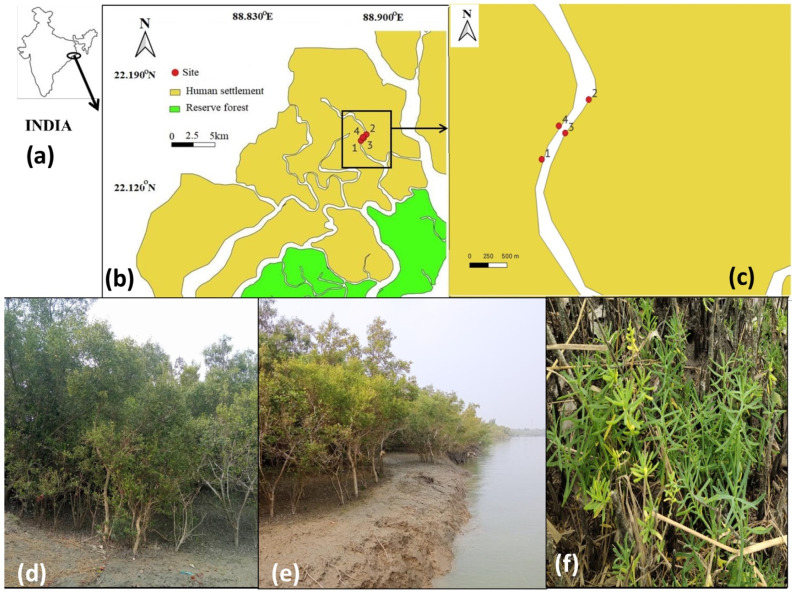
Depicts the quadrat and salinity sampling sites in the central Indian Sundarbans. (**a**) Map of India with the area of Sundarbans zoomed out. (**b**) The whole region of Indian Sundarbans depicted along with the reserve forest and human habitable islands, from which the biodiversity sampling locations (1–4) were zoomed out. (**c**) The biodiversity sampling area zoomed out and depicted as points (1–4). (**d**) The quadrat sampling at the study site with dominance of *Avicennia marina*. (**e**) The sampling site and the adjoining creek in high tide have been depicted in this picture. (**f**) A natural occurrence of *Suaeda maritima* in the study site observed near to the LTL.

The study site has been selected in the central part of the Indian Sundarbans (near Amtali and Chotto-mollakhali revenue villages), as detailed in Figure 1. Located on both sides of a major creek, a natural mangrove patch and its location make it a suitable plot for studying the impact of surface water salinity on the mangrove community ecology. The study site covers a length of 1017 m and a maximum breadth of 164.1 m, spreading from 22°14′39″ N, 88°53′14″ E to 22°15′04″ N, 88°53′36″ E and 22°14′50″ N, 88°53′25″ E to 22°14′53″ N, 88°53′22″ E. The location is an extended mudflat, though near the human settlement, and has limited anthropogenic interventions. Hence, the natural growth of mangroves and the changes in mangrove community ecology can be studied. The area is sheltered on all sides by human-inhabited islands and is about 5 km west of the Jhilla River, which is the natural border between India and Bangladesh. This river also connects to the reserve forests (Sajnekhali Sanctuary, Jhilla Reserve Forest, and Sundarban National Park, India). The sites are on both sides of a creek with an approximate width of 126 m.

### 2.2. Biodiversity Assessment

A temporal study on the mangrove biodiversity was conducted in twelve quadrat plots (10 m × 10 m) and transects (5 m × 20 m) at the study site every year in the month of June between 2017 and 2021. The plant species are noted for reference, and the average individual number of each species was calculated based on the surveyed data following the methods of Curtis and McIntosh [17] and Chowdhury et al. [5]. Through relative density, the composition and richness of the mangrove species were determined.

$$Relative\;Density\;\left(D\right)=\frac{Number\;of\;individuals\;of\;a\;species}{Number\;of\;individuals\;of\;all\;the\;species}\times 100$$

This estimator provides information on a species’ status across the board (i.e., across all species present in the region), which aids in the comparison of that species’ relative positions in the target environment. Simpson’s index for dominance, diversity, and Shannon’s diversity index were used to analyze the effect of the species on the health of the ecosystem, their diversity, and dominance [5]. The ratio between abundance and frequency (A/F) has been estimated to shed light into the distribution pattern of the species [7]. An A/F value < 0.025 indicates ‘regular distribution’, whereas 0.025–0.05 indicates ‘random’, and values > 0.05 indicate ‘contiguous’ distribution pattern.

### 2.3. Salinity Assessment of Tidal Water

Salinity assessment has been studied during the pre-monsoon (May) and post-monsoon (October) seasons at the same site every year from 2017 to 2021. Annual salinity is calculated by taking the mean values of pre- and post-monsoon salinity assessments. During each sampling period in a year, five samples (*n* = 5) have been collected and analyzed. The first sample was collected during the peak of diurnal high tide; the second sample was collected during the peak of diurnal low tide on the same day; the third sample was collected in the night of the first day during the receding tide; the fourth sample was collected during the peak of high tide the next day; and the fifth sample was collected during the second day’s peak low tide. This sampling method was followed to minimize the temporal fluctuations in the salinity results. Changes in the salinity profile in the region are assessed using the Mohr–Knudsen method [5,20]. Then, salinity is calculated from the formula

Salinity (S) = 1.805Cl^−^ + 0.03 g/kg

### 2.4. Soil Sampling and Analyses

Each year from 2017 to 2021, soil samples were collected from the biodiversity sampling location (10 m × 10 m quadrats). The intact cores were persevered in icebox and bought to the lab and homogenized. The bulk samples were physically sorted from the gravel and coarse roots, and the soils were then lightly pulverized with a mortar and pestle. Three replicate subsamples of the sample (10 cm deep) were taken, and then combined and homogenized to generate a composite soil sample. Before being delivered to the lab, the gathered soil samples were put in plastic bags and marked. To remove large pebbles and plant debris, samples were sieved using a 2 mm sieve after being air-dried at ambient temperature (25–30 °C). The core approach was used to calculate soil bulk density.

According to Fang et al., litter primarily affects the top 10 cm of soil depth, and cyclonic influences can be noticed in the topsoil layers [21]. Studies conducted by Castañeda-Moya et al. to understand the impact of Hurricane Wilma in the mangroves of the Florida Coastal Everglades also considered 0–10 cm depth, as the hurricane-transported sediment was observed to impact 0.5–4.5 cm soil depth [22]. 

A multiparameter pH probe (HI-2020, Hanna Instruments, India) was used to measure the pH of the soil. After being shaken and allowed to settle for an hour, soil pH was measured in a suspension of soil and deionized water (1:2.5, *w/v*), and bulk density was calculated according to an established protocol [23]. After digestion with 0.32% KMnO_4_ solution and titration with 0.02 N H_2_SO_4_, the semi-automatic nitrogen estimation system calculated the amount of nitrogen (N) that was available to plants. The Olsen method [24] was used to determine how much phosphorus (P) was available to plants.

### 2.5. Estimation of the Carbon Pool

#### 2.5.1. Elemental Analyzer

To determine soil organic carbon (SOC_EA_) using the elemental analyzer (Euro Vector, EA 3100), the air-dried soils were pretreated with 6 M HCl to eliminate inorganic carbon present in the form of carbonates [25]. The elemental analyzer has been widely used in the carbon estimation of coastal saline or hypersaline soil [26,27].

#### 2.5.2. Wet Digestion Method

Soil organic carbon (SOC_w_) was determined through the wet digestion method [23]. An oxidizing agent (K_2_Cr_2_O_7_ in an acidic H_2_SO_4_ medium) has been used to react with organic matter (reducing agent) present in the soil samples in the presence of a color change indicator [27]. First, 10 milliliters of 0.18 mol L^−1^ of K_2_Cr_2_O_7_ and 20 mL of conc. H_2_SO_4_ were mixed and diluted with 200 mL of distilled water in an Erlenmeyer flask. After filtration, 10 mL of H_3_PO_4_ and 5 drops of diphenylamine were added. The excess Cr_2_O_7_^−2^ was titrated with ammonium iron (II) sulfate with a visible color change from blue to green, and then, it was stoichiometrically correlated with reduced substrate (SOC). As applied by Chowdhury et al., in the analysis of SOC, 5 gm Ag_2_SO_4_ L^−1^ of H_2_SO_4_ was added before the use of H_2_SO_4_ to minimize the interference of Cl^−^ in saline mangrove soil [1]. This Walkley and Black chromic acid wet oxidation method has been widely used by researchers estimating the SOC and the blue carbon stock of mangrove, hypersaline edaphic environments [27,28].

#### 2.5.3. Organic Blue Carbon Pool Assessment

The following calculations were made for the carbon pool (0–10 cm) utilizing SOC_EA_ concentration, bulk density, and specific soil depth [27,29]:

BOC_S_ = SOC_EA_ × BD × T

where BOC_S_ is the blue organic carbon stock in kg C m^−2^, SOC_EA_ is the soil organic carbon estimated by the elemental analyzer in g kg^−^^1^, BD = bulk density (g cm^−^^3^), and T = soil thickness (cm).

### 2.6. Statistical Tests

SPSS 16 (SPSS Inc. Chicago, IL, USA) and Microsoft Excel 2007 have been used for statistical analysis. The trends in temporal changes in salinity regime and biodiversity have been studied using regression analysis. The standard error is used to assess the data’s dependability. Analysis of Variance (one-way ANOVA) has been used to analyze the variation between mean values, followed by Duncan’s post-hoc test. Hierarchical Cluster Analysis (HCA) was used on the species distribution with ‘Ward linkage’ and ‘Squared Euclidian distance’ as the major attributes of determination.

## 3. Result and Discussion

### 3.1. Variations in Tidal Water Salinity Profile

**Figure 2:**
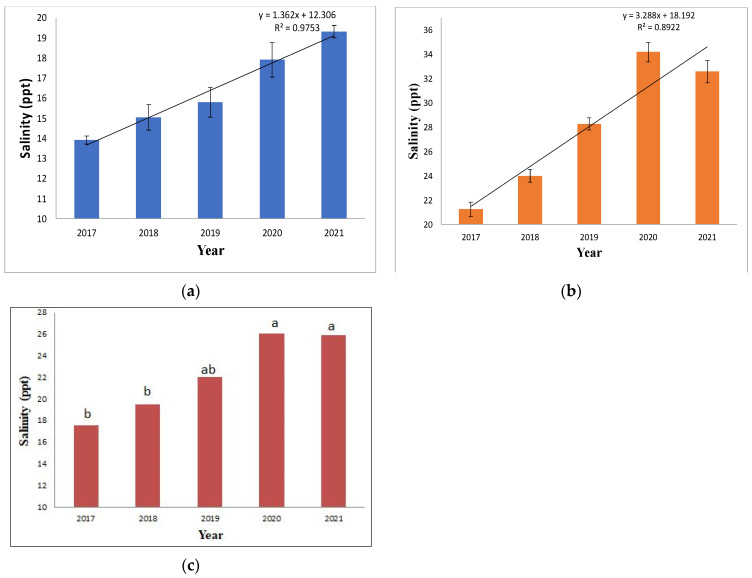
Salinity ranges of surface water analyzed from 2017 to 2021. (**a**) The post-monsoon salinity range (*n* = 5) showed a clear trend of salinity rise from 2017 to 2021. The error bar represents ± 1 standard deviation. (**b**) The pre-monsoon salinity range (*n* = 5) estimated in ppt showed a clear trend of salinity rise. The error bar represents ± 1 standard error. (**c**) The annual change in salinity profile was tested through one-way ANOVA, (*n* = 10); the lowercase alphabetical letters indicate the DMRT post-hoc test.

The result showed a drastic change in the salinity profile in the region owing to cyclone impacts. As evident from Figure 2a, the salinity in the pre-monsoon assessment is higher than the post-monsoon salinity depicted in Figure 2b. The pre-monsoon salinity ranges from 21,240 mg L^−1^ (21.2 ppt) in 2017 to 32,580 mg L^−1^ (32.6 ppt) in 2021. Post-monsoon salinity was far lower than the pre-monsoon estimates, ranging from 13,320 mg L^−1^ (13.3 ppt) in 2017 to 19,300 mg L^−1^ (19.3 ppt) in 2021. The reason behind this is the impact of monsoon rains on the salinity profile of the region. According to previous studies, it is evident that the annual rainfall in the region is high, around 1800 mm y^−1^, of which 80% of precipitation occurs during the monsoon months (July–October) [30]. The regression trend line in both pre-monsoon and post-monsoon estimates shows an increasing salinity trend from 2017 to 2021.

An increasing salinity trend has been observed since 2017, as shown in Figure 2c. The one-way ANOVA test revealed a difference in the yearly means from 2017 to 2021, with the salinity profiles of 2017, 2018, and 2019 showing a similar trend, while those of 2019, 2020, and 2021 show similar trends indicating the cyclone-induced sea water intrusion is there from 2019 to 2021, as per the Duncan post-hoc test followed by ANOVA analysis. The F value was found to be significant with a probability level of 0.017. This firmly indicates that the cyclone induced, increase in salinity in the region. The year 2019 witnessed two cyclones, Fani and Bulbul, while the following years recorded one annual cyclonic event, namely Amphan and Yaas (Table 1). Hence, the ANOVA test depicted in Figure 2c showed 2019 having similar salinity data to previous years—2018, 2017, and coming years impacted by frequent cyclones, i.e., 2020 and 2021. This assessment is in concurrence with the existing literature, which has also indicated an increase in salinity in surface water post-cyclone. The Amphan cyclone was associated with an increase in salinity of ∼1.5 PSU in the impacted region [12]. After the impact of the Fani and Bulbul cyclones, an increase in surface water salinity was observed in the area [11,31]. The Yaas cyclone resulted in increased salinity due to flood water influx in the region, which also impacted agriculture [13].

### 3.2. Variations in Mangrove Diversity

**Figure 3:**
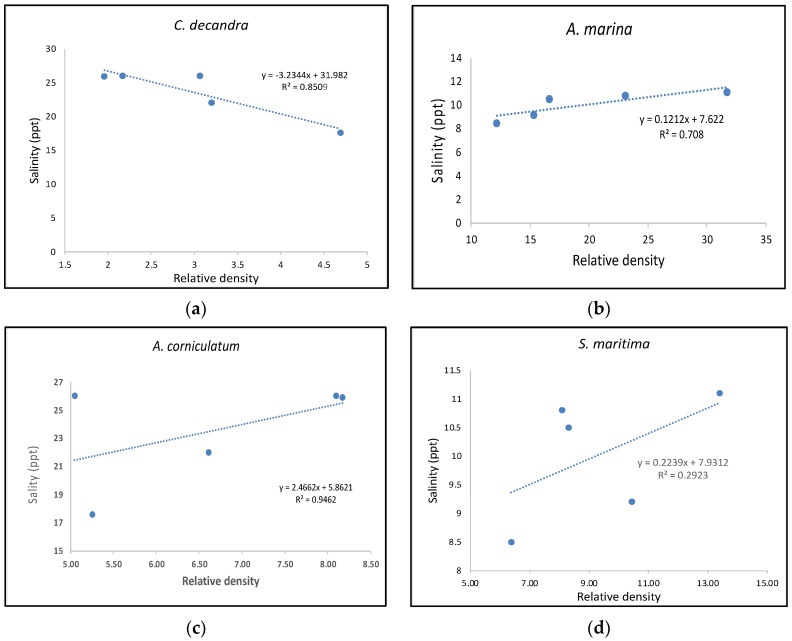
The significant relationship is found between three mangrove species with rising tidal water salinity trends due to the impact of cyclones/storm surges. The figures show that (**b**) *A. marina* and (**c**) *A. corniculatum* are showing a significant rise in relative density with an increase in average salinity from 2017 to 2021, whereas (**a**) *C. decandra*, the true mangrove member of the Rhizophorceae family, show a decreasing relative density with increasing salinity gradient. Mangrove associate (**d**) *S. maritima* has been showing an increasing trend in relative density with salinity with weak correlation.

Previous research has indicated that both *Avicennia marina* and *Aegiceras corniculatum* can tolerate higher salinities than similar other mangrove [32,33,34]. Both are sympatric species with similarities in salinity tolerance, and the former has resilience to cyclone disasters [14,35]. In the current study, Figure 3 shows the relationship between the relative densities of *A. marina*,* A. corniculatum* and *Ceriops decandra* with average annual salinity recorded from 2017 to 2021. Figure 3 indicates that there is an observable positive trend between increases in the number of the former two species with rising salinity, while the last species is a true mangrove of the Rhizophoraceae family, which seems to be sensitive to rapid salinity fluctuations. Previous studies on the impact of salinity on these species show that it is a common trend for reduction of propagule density in *C. decandra* due to rising salinity [14]. *Avicennia alba* showed a drastic reduction in relative density from 13.3 to 1.6 from 2017 to 2021, respectively. *Proteresia corctata* is a Poaceae family member that has been observed colonizing the newly formed mudflats across the Indian Sundarbans. The relative density of the mangrove grass increased from 12.6 to 16 from 2017 to 2021. It is also evident from Table 2 that the relative density of the mangrove grass never dropped below 10, indicating its resilience to the salinity rise induced by the saltwater influx due to cyclones [36]. This indicates that the sedimentation in the mudflat has created an opportunistic ground for the ecosystem to bounce back if the salinity regime is ameliorated in the future [1].

Biodiversity assessment in the region indicates the presence of 16 species in 2017 (Table 2). Relative density reflects a steady reduction in the biodiversity of salt-sensitive species in response increase in salinity. *Xylocarpus granatum*,* Xylocarpus moluccensis*,* Rhizophora mucronata*,* Bruguiera gymnorrhiza*, and *Bruguiera cylindrica* used to have less representation in 2017 but went locally extinct from the studied population by 2021. Previous research on the physiological adaptation of *B. gymnorrhiza* indicated that an increase in salinity results in an augmentation in the evapotranspiration rate of the seedlings [37]. *B. cylindrica* is a sympatric species like *B. gymnorrhiza*.* X. granatum *and* X. moluccensis* share a similar niche and position in the Sundarbans mangrove ecosystem. *X. granatum* seedlings show greater growth in freshwater in comparison to higher salinity (around 23 ppt), as per a previous study conducted in Micronesia [38]. Following a similar trend, the species showed extinction in the studied population when the salinity rose to around 26 ppt in 2020 due to the Amphan super cyclone. Even without salinity-increasing impacts, these species showed smaller population proportions (relative density = 0.94 for *X. granatum* and relative density = 0.56 for *X. moluccensis*) in 2017, and when salinity stress was impacted due to cyclones, the regeneration of the seedling became difficult due to seedling mortality. Hence, the population would be extinct by 2021. *R. mucronata* shows tolerance to salinity fluctuations by increasing xylary vessel density [39]. However, if there is a rapid fluctuation in salinity, it may not have enough time to adjust to the changing abiotic factors. The Rhizophoraceae (true mangrove) member has a very small population; beginning in 2017, the population could not adapt significantly to adjust to the changing salinity pattern and became extinct in the regional population. Previous studies have already shed light on the proneness of rare plant species to go extinct in the case of abiotic changes or sudden random events [40]. A similar case was noticed where *R. mucronata* dominated shorelines, resulting in 100% mortality due to the impact of Cyclone Eline [41]. This indicates that this species is less resilient to cyclone stress. *Sonneretia caseolaris* showed a complete extinction in the population until 2020, which may be due to the Amphan super-cyclone-induced flushing of saltwater in the inner estuaries. However, as per previous estimates, there are populations of *S. caseolaris* in the eastern part of the Indian Sundarbans, and the propagules floating through the creek may have settled and started a natural regeneration during 2021. Previous studies also indicated the resilience of this species during instances of cyclones or storm surges [42]. An increase in the relative density of *Excoecaria agallocha* from 5.6 in 2017 to 10.87 in 2019 and a reduction back to 7.52 in 2021 indicated a variable but salinity-resilient response of this mangrove associate. Studies conducted at other mangrove habitats under cyclone impact show the resilience of this plant [39]. *Phoenix padulosa* is generally observed in degraded mudflats as an edaphic sub-climax species [5,43]. In the current study, this species shows a decreasing distribution across the years, with a relative density of 3 in 2017 dropping to 1.63 in 2021. Figure 3 shows the relationship between relative density and salinity rise.

**Figure 4:**
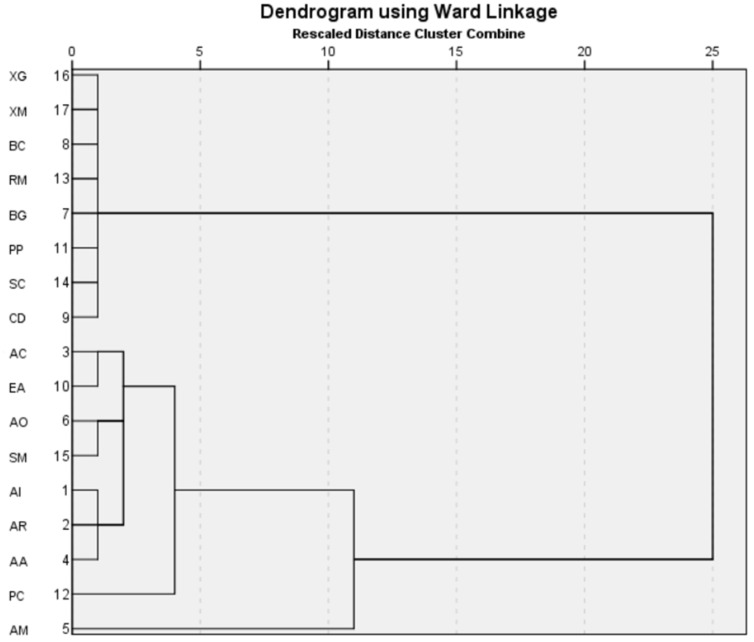
HCA indicates the association between the species between 2017 and 2021. The abbreviation of the species is as noted in Table 2.

Overall, the study indicates an increasing dominance of salinity-resilient *A. marina* over the years, which is one of the most resilient mangroves [5,44]. HCA has been used to find associations between the species across the years (Figure 4).

The HCA indicates the same association as is evident from the visual and species diversity analysis. *A. marina* was found to be distributed without any specific association with other species, which is probably due to its high salinity tolerance. Species such as *X. granatum*,* X. moluccensis*,* R. mucronata*,* B. gymnorrhiza*,* B. cylindrica*,* R. mucronata*,* C. decandra*,* S. caseolaris*, and *P. padulosa*, showed similar associations due to their decreasing diversity because of the salinity rise induced by cyclones. These species are under the maximum threat of local extinction due to the cyclone-induced salinity rise. *P. coarctata*: as the pioneer species in the mudflat mangrove succession, it is seen to be distantly associated with species that can tolerate and survive the increasing salinity regime, and hence is closely clustered with species that can tolerate the cyclonic disturbances.

This is also evident with the increasing value of Simpson’s Index of Dominance from 0.08 in 2017 to 0.16 in 2021 (Table 3). *S. maritima* is a mangrove associate herb with high salinity tolerance and is mostly seen near the low tide level in salt-encrusted mudflats, as evident in previous study [5]. This mangrove associate is showing a weak but positive linear correlation with the rise in annual salinity gradient of tidal water. This indirectly impacted the diversity of the meta-population, as the dominance index is reciprocal to diversity estimation; hence, more dominance indicates lesser diversity [5,45]. Similarly, as Simpson indexes are biased toward dominant species, the information statistic index of the Shannon Index of Diversity reflects the overall health of the ecosystem, considering the rarer species in the equation (Table 3) [45]. The Shannon index generally ranges from 1.5 to 3.5 in a natural ecosystem, and it reflects the overall health of the ecosystem. Table 3 indicated a gradual decline in ecosystem health from 2017 to 2021, with Shannon index values of 2.51 to 2.06. This shows a declining diversity and increasing dominance of salinity-tolerant mangrove species.

**Figure 5:**
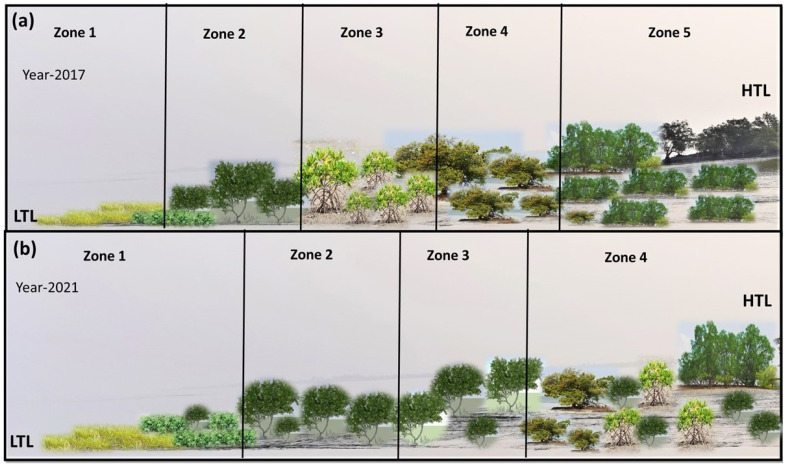
The changes in zonation pattern have been observed in the study site between 2017 and 2021. (**a**) Before the cyclonic disturbances (2017), the study area was divided into five zones, where Zone 1 has a predominance of *Suaeda maritima* and *Proteresia coactata*, followed by Zone 2 with *Avcennia marina*, *Acanthus ilicifolius* and *Aegiceras corniculatum*, followed by *Avicennia alba, Avicennia officinalis and Rhizophora mucronata* Zone 3. Zone 4 is mostly dominated by *Aegialitis rotundifolia and Ceriops decandra*, while the last zone (Zone 5) has tree species such as *Sonneretia caseolaris*, *Xylocarpus granatum, Xylocarpus moluccensis* and *Excoecaria agallocha.* (**b**) Phytosociological assessment of the study area after consecutive cyclone instances and a rise in salinity (2021) indicated a shift in the community composition in the zones. Zone 1 is again predominated by *Suaeda maritima* and *Proteresia coactata* followed by *Avicennia marina* and *Acanthus ilicifolius* in Zone 2. Zone 3 has an association of *Avicennia marina* and *Aegiceras corniculatum* followed by *Avicennia officinalis* and *Avicennia alba*, while Zone 5 was not found to be distinctly identifiable. Hence, it is seen that mangrove zonations in response to tidal amplitude (from LTL to HTL) are becoming homogeneous with a dominance of *A. marina* in most of the associations.

A phytosociological study also revealed a change in the zonation pattern at Indian Sundarbans, which is depicted in Figure 5.

Previous research has highlighted the presence of zonation at the Indian Sundarbans from low tide level (LTL) to high tide level (HTL) [46,47]. In the present study, a similar zonation was observed in the study site. However, over the period of 5 years, *A. marina* started to dominate both Zones 2 and 3, while the true mangrove tree species was replaced by *A. alba*,* A. officinalis*, and *C. decandra* in Zone 4. The zones are becoming more and more homogeneous with an overall dominance of *A. marina*. During the 2017 assessment, A. marina was seen to be dominating in Zone 2, whereas in 2021, it was found to be dominating in both Zones 2 and 3, whereas Zone 5 was not observed, which was unlike in the 2017 assessment. The A/F value noted in Table 2 indicates a ‘contiguous’ (>0.05) distribution pattern for *S. maritima*, *P. corctata*, *A. marina*, *E. agallocha*, *A. ilicifolius*, and *A. corniculatum* between 2017 and 2021, but for *C. decandra*, the distribution pattern changed from ‘contiguous’ in 2017 to ‘random’ in 2021 (i.e., 0.025–0.05). Species such as *X. granatum*,* X. moluccensis*,* R. mucronata*,* B. gymnorrhiza*, and *B. cylindrica* showed a ‘regular’ (< 0.025) distribution in 2017 but became locally extinct by 2021. This indicates that these species are sensitive to random environmental fluctuations due to the cyclones and need further research on identifying the best restoration techniques.

### 3.3. Changes in Carbon Pool and Soil Parameters

The concentrations of soil organic carbon, soil salinity, available nitrogen, and available phosphorus and the nutrient cycling in sediments can be critical decisive factors controlling the density and development of mangrove forests. The growth pattern of mangrove forest is highly dependent on the above-mentioned abiotic factors. However, the change in the mangrove biodiversity also plays a crucial role in the change in these soil parameters as evident from earlier studies [48,49]. Thus, the changes in biodiversity have an impact on ecological services.

**Figure 6:**
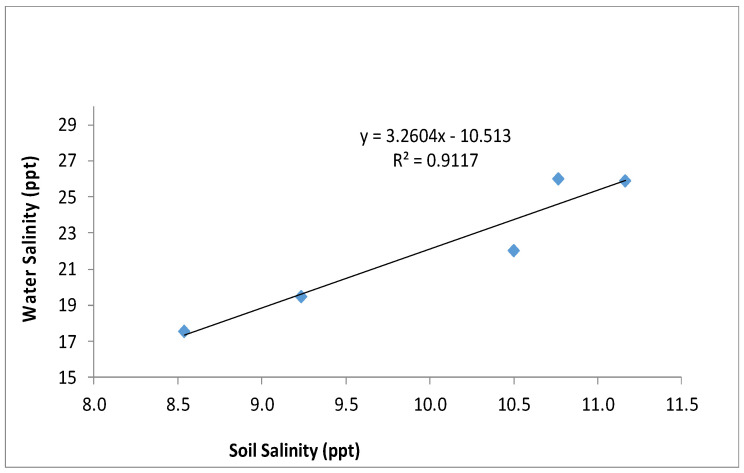
The relationship between average annual tidal water salinity and annual soil salinity between 2017 and 2021. For easy comparison, both the salinity units were taken as parts per thousand (ppt).

Research has shown a decrease in blue carbon pools with degrading or deteriorating ecological functions and biodiversity [34,50]. Table 4 elucidates the changes in soil chemical constituents at the sampling site during the study period. The pH and bulk density do not show significant changes with the year, but one-way ANOVA indicates a significant temporal variation in SOC_EA_, SOC_W_, available nitrogen, and available phosphorous. Soil salinity significantly increased from 2019 onwards after the Fani, Bulbul, Amphan and Yaas cyclones hit the region. However, a slight increase in salinity was there already in 2018. The post-hoc test is also indicating the similarity between the salinity in year 2017 and 2018 and between 2019 and 2021. Soil salinity is also dependent on tidal water salinity, which is evident from Figure 6.

The concentrations of available N and available P were recorded as being approximately 1.5 times lower in 2021 in comparison to 2021 (Table 4). The gradual decrease in the concentration of the available N and the P with increasing soil salinity indicates the impact of salinity on the micro-organisms as well as on the reducing mangrove density. The cyclone Fani hit west Bengal in 2019, and a significant decrease in SOC, available N and available P can be seen in Table 4. Previous studies also suggested that that the concentration of organic C, available N and P was lower in the disturbed sites, which indicates the lower microbial activities [34].

**Figure 7:**
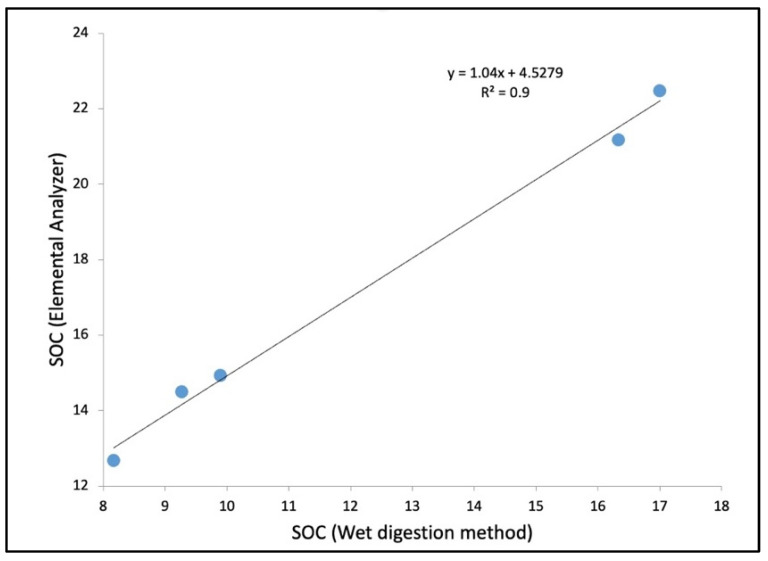
The linear correlation between the values of SOC obtained through the wet digestion method (SOC_W_) and by the elemental analyzer method (SOC_EA_). The units considered for both variables are g/kg.

SOC_W_ is prone to interferences due to manual error, while titration or changing strength of K_2_Cr_2_O_7_ during analysis can influence the outcome. An elemental analyzer has been widely used in the carbon estimation of coastal saline or hypersaline soil [25,26,27]. Although a positive relationship was observed between the concentration of SOC_W_ and SOC_EA_ with a ‘R^2^’ value of 0.9 (Figure 7), a lower SOC_W_ value has been observed in comparison with the SOC_EA_ value, which may be due to interferences or manual errors while conducting the wet digestion analysis. As evident from work of Passos et al. and Nóbrega et al., C estimation with an elemental analyzer has better accuracy in case of coastal and mangrove soil [26,27].

**Figure 8:**
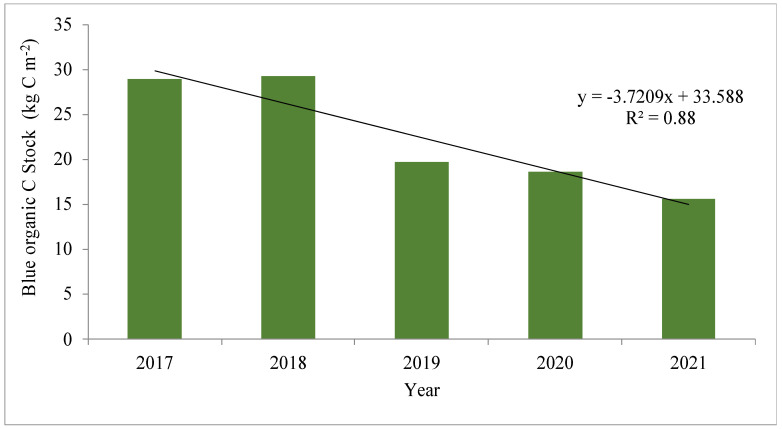
The changes in organic carbon pool (kg C m^−2^) at the study site between 2017 and 2021. The SOC_EA_ has been used to compute the blue organic carbon stock.

Blue carbon sequestration is one of the key services of mangrove forests [1]. The changes in biodiversity patterns do impact the carbon pool, as evident from Figure 8. It indicates a steady decline in BOC_S_ from 2017 to 2021 with a negative correlation with years and an ‘R^2^’ value of 0.88. A rise in soil salinity or electrical conductivity is coinciding with cyclonic disturbances in the area. Higher salinity can change the nitrogen dynamics of soil, which may be the reason for lower N over the years in the present study [51].

The work of Chambers et al. also highlights an increase in dissolved organic carbon (DOC) in the mangrove soil pore water due to increased inundation and salinity rise [52]. An increase in DOC means more allochthonous transport to a distant location and less accretion of carbon in the mudflat.

*A. marina* residing along the fringe of the forest has a leaf carbon content of 39.1%, while species such as *B. gymnorrhiza* (45.6% C), *E. agallocha* (44.8% C), *R. mucronata* (41.2% C), and *X. granutum* (40.7% C) have a higher carbon content in their leaves [53,54]. The mangrove ecosystem is evergreen. Most of the energetics of the ecosystem is managed through the detritus food chain and the decomposition of nutrients in the leaves. With low microbial diversity, it takes more time for the waxy mangrove leaves to be decomposed in the soil and release carbon and nutrients. The salinity-tolerant *A. marina* has lower carbon content in the leaves, resulting in low carbon sequestration in the soil in comparison with a diverse ecosystem with other species. Salinity also impacts the biodiversity of microbes and hence can alter the time required for releasing nutrients back to the soil, including C, in the case of an increased salinity increment.

This study indicated a lower concentration of the major macromolecules in soil, proving a degradation of ecological functions and services that can be linked with changes in mangrove community ecology in response to natural disaster-induced salinity intrusions. The work of Mace et al. also indicates a relationship between ecosystem structure, function, and services [55].

## 4. Cyclones, Salinity Intrusions and Impact on Global Blue Carbon Sink

Global greenhouse gas emissions are responsible for trapping heat in the atmosphere. In a positive feedback loop, this entrapment of heat results in a rise in the overall oceanic temperature and accelerates the surface evaporation process. The accelerated evaporation cycle in the tropical oceans has increased the frequency and intensity of cyclones in the last decade. In the Indian Ocean and nearby oceanic regimes, the temperature is warming the oceans, disrupting the general oceanic circulations, and increasing the risk of the development of tropical disturbances or cyclones [56,57,58]. Similarly, the Bay of Bengal has also been impacted by the global problems of climate change and global warming. El Niño Southern Oscillation (ENSO) has been proven to also influence the Indian Ocean dipole and influence cyclogenesis throughout the southern oceans [58]. Kang and Elsner estimated an average global cyclone intensity of 1.3 m s^−2^ over the last 30 years due to global warming impacts [2]. Although predictive models are present, it is difficult to comprehend the impact of climate change or global warming at regional levels, particularly in coastal ecosystems. Saltwater flows in through the tides, while the freshwater flow is maintained due to riverine transport in a deltaic or estuarine system.

Cyclones destabilize ecosystems. As per previous research, cyclones and natural disasters are one of the main reasons behind declining mangrove cover across the globe [59]. Due to the sediment load of the Ganga, Brahmaputra, and Meghna River systems, along with the earth’s movement some 200 years ago, the Bengal Basin is tilting eastward. Recent trends indicate a reduction of freshwater from Himalayan rivers coupled with an increase in sea level impacting the Sundarban mangrove ecosystem [60]. This geological reason can also be responsible for increased salinity in the delta by facilitating seawater intrusion. Around 123 countries located in the tropical and subtropical regions of the globe have mangrove vegetation along their coastlines, estuaries, or deltas [61]. Mangrove ecosystems are regarded as one of the best natural models to study the impact of climate change and global warming-related vagaries such as rises in salinity and sea level and cyclone impacts, and they give a glimpse into understanding the impact of these global problems at regional levels [62]. Hence, research on understanding climate change and natural disaster impacts on mangrove biodiversity also paves the way to understanding the impact of these global problems on different ecosystems across the world. Blue carbon sequestration by mangroves and sea grasses plays a crucial role in the fight against climate change (SDG 13), and a healthy ecosystem conserves the species diversity in the wetlands (SDG 14). It is observed by scientists that mangroves have the potential to withstand the vagaries of climate change and sea level rise according to Menéndez et al., but this resilience may have a limit and will eventually result in irreparable changes in mangrove community structure and composition, as is also evident from this study [63].

## 5. Conservation Consideration and Recommendation

The Indian part of the Sundarbans comprises 102 islands, out of which 54 are inhabited. The rest of the islands are under mangrove cover. The river Ganges carries down 262million tons of sediment per year, but research indicates that only 7% is diverted through its southern distributaries [64]. The low discharge restricts this sediment in the mangrove forested islands. It creates a unique mud island diurnally washed by tides and soil consolidated by mangrove roots. It is recognized as the 27th Ramsar wetland site of India in 2019, making it the largest wetland in the nation. Ecotourism is booming in the region. There has been a rise in ecotourism income (INR) from 1,508,000 in 2003–2004 to 11,772,000 between 2012 and 2013 [65].

Mangroves hold sediment, and their long-extended rhizosphere helps in consolidating sediment during storm surges and cyclonic disturbances. As per previous studies, mangroves have acted as a bio-shield against natural disasters along the coastlines by acting as a wind break, consolidating soil, reducing wave force, and protecting the coastlines [47,59,63]. Although recent research has focused on the importance of blue carbon and disaster resilience ecosystem service of mangroves for mitigating climate change threats, the ecological investigations on the climate change impacts on mangrove communities are less explored [66,67]. Friess et al. opined that a rise in temperature can destabilize the ecological function and services of global mangrove ecosystem, substantiating a research focus on the climate change–mangrove community ecology nexus [67]. Segaran et al. have indicated that the publication trends in this domain mostly focus on carbon sequestration, nature-based solutions, and disaster resilience, but few works focus on ecological changes in the mangrove community due to climate change vagaries [66]. This necessitates the need for focused ecological research to formulate effective solutions to tackle the issue.

The solution lies in the protection of the areas that are outside the reserve forest and the restoration of mudflats with multispecies mangroves [1,68]. These meta-populations also act as a natural repository of propagules and are fundamental for a sustainable ecosystem by facilitating the colonization of newly formed or degraded mudflats [69,70]. Thus, the changes in community structure can also reduce the availability of propagules of salinity-sensitive mangrove species; hence, they will impact the overall biodiversity of the ecosystem in the unforeseen future.

This research sheds light on the impact of salinity rises in surface water on mangrove ecological composition and services, which can hinder achieving the goals of SDG 13 and 14. Hence, ecological engineers need to comprehend the role of mangrove biodiversity in its carbon sequestration potential. According to this case study, a 45% reduction in the blue carbon pool is observed due to a 47.6% increase in surface water salinity from 2017 to 2021. This study highlights the negative impact of salinity intrusion on blue carbon sequestration as well as mangrove biodiversity. A similar situation may exist at mangrove patches scattered across the globe and requires a similar investigation.

## 6. Conclusions

The research proved the increase in surface salinity trend due to cyclones in the inner-central part of the Indian Sundarbans. This is resulting in the dominance of salinity-tolerant mangrove species while causing localized extinctions of salinity-sensitive members. It is also negatively impacting the health of the ecosystem and can disrupt the ecological services of the ecosystem. The changes in community composition are associated with a reduction in organic blue carbon sequestration by mangrove soil along with reduction in other major nutrients such as available nitrogen and phosphorus. This study indicates a deterioration of ecological health, diversity, changes in mangrove species zonation in response to tidal gradient and functions due to a sudden change in the surface water salinity profile due to natural disturbances. Other causes of salinity rise, such as sea level rise, climate change, and a reduction in freshwater flow in the delta, can also contribute to increased salinity, which needs further investigation. Future research focus needs to be directed toward establishing the connection between climate change, global warming, cyclogenesis, and changes in estuarine salinity profiles to formulate any future conservation policies for coastal habitats. Conservation plans also need to be put in place to manage this localized extinction, as it can destabilize the entire Sundarban mangrove ecosystem.

## Figures and Tables

**Table 2:** Relative density (RD) and A/F ratio of mangrove species in the sample site between 2017 and 2021.

Species	Family	Abbreviation	2017		2018		2019		2020		2021	
			RD	A/F	RD	A/F	RD	A/F	RD	A/F	RD	A/F
*Acanthus ilicifolius* L.	Acanthaceae	AI	9.38	0.09	9.4	0.09	6.82	0.07	8.49	0.08	5.23	0.08
*Aegialitis rotundiflia* Roxb.	Plumbaginaceae	AR	9.94	0.10	10.3	0.10	9.59	0.10	7.31	0.07	5.56	0.06
*Aegiceras corniculatum* (L.) Blanco	Primulaceae	AC	5.25	0.05	5.0	0.05	6.61	0.07	8.09	0.08	8.17	0.08
*Avicennia alba* Blume	Acanthaceae	AA	13.32	0.13	11.4	0.11	8.10	0.08	10.27	0.10	1.63	0.05
*Avicennia marina* (Forssk.) Vierh.	Acanthaceae	AM	12.20	0.12	15.3	0.15	16.63	0.17	23.10	0.23	31.70	0.32
*Avicennia officinalis* L.	Acanthaceae	AO	6.00	0.06	9.0	0.09	7.89	0.08	10.46	0.10	6.54	0.07
*Bruguiera gymnorrhiza* (L.) Lam.	Rhizophoraceae	BG	4.50	0.05	1.8	0.02	1.07	0.02	1.18	0.04	0.00	-
*Bruguiera cylindrica* (L.) Blume	Rhizophoraceae	BC	0.75	0.01	0.2	0.01	0.00	-	0.00	-	0.00	-
*Ceriops decandra* (Griff.) Ding Hou	Rhizophoraceae	CD	4.69	0.05	3.1	0.03	3.20	0.03	2.17	0.03	1.96	0.02
*Excoecaria agallocha* L.	Euphorbiaceae	EA	5.63	0.06	4.5	0.05	10.87	0.11	6.32	0.06	7.52	0.08
*Phoenix padulosa* Roxb.	Arecaceae	PP	3.00	0.03	2.0	0.02	2.13	0.03	1.18	0.04	1.63	0.05
*Proteresia corctata* (Roxb.) Tateoka	Poaceae	PC	12.57	0.13	12.6	0.13	13.43	0.13	11.85	0.12	16.01	0.16
*Rhizophora mucronata* Lam.	Rhizophoraceae	RM	0.56	0.01	0.4	0.01	1.18	0.01	1.38	0.02	0.00	-
*Sonneretia caseolaris* (L.) Engl.	Lythraceae	SC	4.32	0.04	4.0	0.04	3.62	0.04	0.00	-	0.65	0.02
*Suaeda maritima* (L.) Dumort.	Amaranthaceae	SM	6.38	0.06	10.5	0.10	8.32	0.08	8.09	0.08	13.40	0.13
*Xylocarpus granatum* K.D. Koenig	Meliaceae	XG	0.94	0.01	0.5	0.01	0.21	0.01	0.00	-	0.00	-
*Xylocarpus moluccensis* K.D. Koenig	Meliaceae	XM	0.56	0.01	0.2	0.01	0.21	0.01	0.00	-	0.00	-

**Table 3:** Changes in diversity dominance and information that statistic indices between 2017 and 2021.

Year	2017	2018	2019	2020	2021	Formula
Shannon Index of Diversity	2.561	2.45	2.451	2.294	2.064	$H\prime =-\sum_{i=1}^{s}piLn\;pi$
Simpson’s Dominance	0.083	0.094	0.093	0.114	0.159	$D=\sum_{i=1}^{s}\frac{n\left(n-1\right)}{N\left(N-1\right)}$
Simpson’s Diversity	0.917	0.906	0.907	0.886	0.841	L=1−D

**Table 4:** Variations in soil quality parameters of the study area from 2017 to 2021.

Year	pH	SOC_W_ (%)	SOC_EA_ (g kg^−1^)	BD (g/cm^3^)	Available N (mg kg^−1^)	Available P (mg kg^−1^)	Salinity (ppt)
2017	7.2 ± 0.15	0.82 ^a^ ± 0.12	21.1 ^b^ ± 1.6	1.37 ± 0.15	75.9 ^b^ ± 6.28	6.17 ^b^ ± 0.35	8.54 ^b^ ± 0.45
2018	7.3 ± 0.21	0.93 ^a^ ± 0.16	22.4 ^b^ ± 1.9	1.30 ± 0.10	74.63 ^b^ ± 7.97	6.13 ^b^ ± 0.29	9.23 ^b^ ± 0.87
2019	7.3 ± 0.10	0.99 ^a^ ± 0.19	14.9 ^a^ ± 2	1.32 ± 0.03	71.13 ^b^ ± 6.86	4.87 ^a^ ± 0.70	10.5 ^b^ ± 0.70
2020	7.4 ± 0.12	1.70 ^b^ ± 0.17	14.5 ^a^ ± 1.4	1.28 ± 0.10	54 ^a^ ± 8.11	4.37 ^a^ ± 0.32	10.77 ^a^ ± 1.42
2021	7.5 ± 0.15	1.63 ^b^ ± 0.15	12.6 ^a^ ± 1.4	1.23 ± 0.25	52.83 ^a^ ± 7.35	4.13 ^a^ ± 0.35	11.17 ^a^ ± 1.35
F value	1.426	**20.91**	**21.13**	0.336	**7.17**	**15.08**	**3.49**

The letters in the superscripts indicate the DMRT post-hoc test, and the significant ‘F’ results (with significance bellow 0.5) have been put in bold.

## Data Availability

Data is with the authors and will be provided upon request.

## References

[1] Chowdhury A., Naz A., Bhattacharyya S., Sanyal P. (2018). Cost–benefit analysis of ‘Blue Carbon’ sequestration by plantation of few key mangrove species at Sundarban Biosphere Reserve, India. Carbon Manag..

[2] Kang N.Y., Elsner J.B. (2015). Trade-off between intensity and frequency of global tropical cyclones. Nat. Clim. Chang..

[3] Friess D.A., Webb E.L. (2014). Variability in mangrove change estimates and implications for the assessment of ecosystem service provision. Glob. Ecol. Biogeogr..

[4] DasGupta R., Shaw R. (2017). Perceptive insight into incentive design and sustainability of participatory mangrove management: A case study from the Indian Sundarbans. J. For. Res..

[5] Chowdhury A., Sanyal P., Maiti S.K. (2016). Dynamics of mangrove diversity influenced by climate change and consequent accelerated sea level rise at Indian Sundarbans. Int. J. Glob. Warm.

[6] Asbridge E., Lucas R., Accad A., Dowling R. (2015). Mangrove response to environmental changes predicted under varying climates: Case studies from Australia. Curr. For. Rep..

[7] Curtis J.T., Cottom G. (1956). Plant ecology workbook. Laboratory field production in Andropogan gerardi. Ecology.

[8] Hoppe-Speer S.C., Adams J.B., Rajkaran A., Bailey D. (2011). The response of the red mangrove Rhizophora mucronata Lam. to salinity and inundation in South Africa. Aquat. Bot..

[9] Gong W., Shen J., Reay W.G. (2007). The hydrodynamic response of the York River estuary to Tropical Cyclone Isabel, 2003. Estuar. Coast Shelf Sci..

[10] Xiao Y., Wang X.H., Ritchie E.A., Rizwi F., Qiao L. (2019). The development and evolution of the Burdekin River estuary freshwater plume during Cyclone Debbie (2017). Estuar. Coast Shelf Sci..

[11] Paul S., Karan S., Bhattacharaya B.D. (2020). Effects of cyclone Fani on the copepod community of the Ganges River estuary of India. Environ. Monit. Assess..

[12] Chacko N., Jayaram C. (2022). Response of the Bay of Bengal to super cyclone Amphan examined using synergistic satellite and in-situ observations. Oceanologia.

[13] Khatun M., Garai S., Sharma J., Singh R., Tiwari S., Rahaman S.M. (2022). Flood mapping and damage assessment due to the super cyclone Yaas using Google Earth Engine in Purba Medinipur, West Bengal, India. Environ. Monit. Assess..

[14] Chowdhury A., Naz A., Bhattacharryya S., Sanyal P. (2021). Dynamics of salinity intrusion in the surface and ground water of Sundarban Biosphere Reserve, India. IOP Conf. Ser. Earth Environ. Sci..

[15] Paul S., Chowdhury S. (2021). Investigation of the character and impact of tropical cyclone Yaas: A study over coastal districts of West Bengal, India. Saf. Extrem. Environ..

[16] Bianchi T.S., Allison M.A., Zhao J., Li X., Comeaux R.S., Feagin R.A., Kulawardhana R.W. (2013). Historical reconstruction of mangrove expansion in the Gulf of Mexico: Linking climate change with carbon sequestration in coastal wetlands. Estuar. Coast Shelf Sci..

[17] Curtis J.T., McIntosh R.R. (1950). The interrelations of certain analytic and synthetic phytosocialogical characters. Ecology.

[18] Hoque M.E., Arafat M.Y., Uddin M.N., Alam H.M.E., Ahmed K.T. (2020). Rapid assessment of SST and Chlorophyll concentration variability due to cyclone Bulbul in the Bay of Bengal using remotely sensed satellite image data. Authorea Preprints.

[19] Chakma P., Akter A. (2021). Flood Mapping in the Coastal Region of Bangladesh Using Sentinel-1 SAR Images: A Case Study of Super Cyclone Amphan. In J. Civil Eng. Forum.

[20] Strickland J.D.H., Parson T.R. (1972). A practical handbook of sea-water analysis. J. Fish. Res. Board Can..

[21] Fang X.M., Wang G.G., Xu Z.J., Zong Y.Y., Zhang X.L., Li J.J., Chen F.S. (2021). Litter addition and understory removal influenced soil organic carbon quality and mineral nitrogen supply in a subtropical plantation forest. Plant Soil.

[22] Castañeda-Moya E., Twilley R.R., Rivera-Monroy V.H., Zhang K., Davis S.E., Ross M. (2010). Sediment and nutrient deposition associated with Hurricane Wilma in mangroves of the Florida Coastal Everglades. Estuaries Coast..

[23] Maiti S.K. (2013). Ecorestoration of the Coalmine Degraded Lands.

[24] Olsen S.R. (1954). Estimation of Available Phosphorus in Soils by Extraction with Sodium Bicarbonate.

[25] Bisutti I., Hilke I., Raessler M. (2004). Determination of total organic carbon—An overview of current methods. Trends Anal Chem..

[26] Nóbrega G., Ferreira T.O., Artur A.G., Mendonca E., Leão R.A.D.O., Teixeira A., Otero X.L. (2015). Evaluation of methods for quantifying organic carbon in mangrove soils from semi-arid region. J. Soils Sediments.

[27] Passos T.R., Artur A.G., Nobrega G.N., Otero X.L., Ferreira T.O. (2016). Comparison of the quantitative determination of soil organic carbon in coastal wetlands containing reduced forms of Fe and S. Geo-Mar. Lett..

[28] Rozainah M.Z., Nazri M.N., Sofawi A.B., Hemati Z., Juliana W.A. (2018). Estimation of carbon pool in soil, above and below ground vegetation at different types of mangrove forests in Peninsular Malaysia. Mar. Pollut. Bull..

[29] Howard J., Hoyt S., Isensee K., Pidgeon E., Telszewski M. (2014). Coastal Blue Carbon: Methods for Assessing Carbon Stock and Emission Factors in Mangroves, Tidal Salt Marshes, and Seagrasses.

[30] Mandal U.K., Nayak D.B., Mullick S., Samui A., Jana A.K., Mahanta K., Raut S., Roy S., Burman D. (2019). Trend analysis of weather parameters over Indian Sundarbans. J. Agrometeorol..

[31] Chauhan A., Singh R.P., Dash P., Kumar R. (2021). Impact of tropical cyclone “Fani” on land, ocean, atmospheric and meteorological parameters. Mar. Pollut. Bull..

[32] Ball M.C. (1988). Salinity tolerance in the mangroves *Aegiceras corniculatum* and *Avicennia marina*. I. Water use in relation to growth, carbon partitioning, and salt balance. Funct. Plant Biol..

[33] Nguyen H.T., Meir P., Sack L., Evans J.R., Oliveira R.S., Ball M.C. (2017). Leaf water storage increases with salinity and aridity in the mangrove *Avicennia marina*: Integration of leaf structure, osmotic adjustment and access to multiple water sources. Plant Cell Environ..

[34] Chowdhury R., Sutradhar T., Begam M.M., Mukherjee C., Chatterjee K., Basak S.K., Ray K. (2019). Effects of nutrient limitation, salinity increase, and associated stressors on mangrove forest cover, structure, and zonation across Indian Sundarbans. Hydrobiologia.

[35] Ball M.C. (2002). Interactive effects of salinity and irradiance on growth: Implications for mangrove forest structure along salinity gradients. Trees.

[36] Latha R., SrinivasRao C., Sr Subramanium H.M., Eganathan P., Swaminathan M.S. (2004). Approaches to breeding for salinity tolerance—A case study on *Porteresia coarctata*. Ann. Appl. Boil..

[37] Takemura T., Hanagata N., Sugihara K., Baba S., Karube I., Dubinsky Z. (2000). Physiological and biochemical responses to salt stress in the mangrove, *Bruguiera gymnorrhiza*. Aquat. Bot..

[38] Allen J.A., Krauss K.W., Hauff R.D. (2003). Factors limiting the intertidal distribution of the mangrove species *Xylocarpus granatum*. Oecologia.

[39] Schmitz N., Verheyden A., Beeckman H., Kairo J.G., Koedam N. (2006). Influence of a salinity gradient on the vessel characters of the mangrove species *Rhizophora mucronata*. Ann. Bot..

[40] Levin D.A., Francisco-Ortega J., Jansen R.K. (1996). Hybridization and the extinction of rare plant species. Conser. Boil..

[41] Macamo C.C.F., Massuanganhe E., Nicolau D.K., Bandeira S.O., Adams J.B. (2016). Mangrove’s response to cyclone Eline (2000): What is happening 14 years later. Aquat. Bot..

[42] Aung T.T., Mochida Y., Than M.M. (2013). Prediction of recovery pathways of cyclone-disturbed mangroves in the mega delta of Myanmar. For. Ecol. Manag..

[43] Badola R., Hussain S.A. (2005). Valuing ecosystem functions: An empirical study on the storm protection function of Bhitarkanika mangrove ecosystem, India. Environ. Conserv..

[44] Patel N.T., Gupta A., Pandey A.N. (2010). Salinity tolerance of *Avicennia marina* (Forssk.) Vierh. from Gujarat coasts of India. Aquat. Bot..

[45] Smith R.L., Smith T.M., Hickman G.C., Hickman S.M. (1998). Elements of Ecology.

[46] Naskar S., Palit P.K. (2015). Anatomical and physiological adaptations of mangroves. Wetl. Ecol. Manag..

[47] Chowdhury A., Naz A., Dasgupta R., Maiti S.K. (2022). Blue Carbon: Comparison of Chronosequences from *Avicennia marina* Plantation and Proteresia coarctata Dominated Mudflat, at the World’s Largest Mangrove Wetland. Sustainability.

[48] Lo’pez-Hoffman L., Anten N.P.R., Martinez-Ramos M., Ackerly D.D. (2007). Salinity and light interactively affect neotropical mangrove seedlings at the leaf and whole plant levels. Oecologia.

[49] Lovelock C.E., Ball M.C., Choat B., Engelbrecht B.M., Holbrook N.M., Feller I.C. (2006). Linking physiological processes with mangrove forest structure: Phosphorus deficiency limits canopy development, hydraulic conductivity and photosynthetic carbon gain in dwarf Rhizophora mangle. Plant Cell Environ..

[50] Rahman M.M., Zimmer M., Ahmed I., Donato D., Kanzaki M., Xu M. (2021). Co-benefits of protecting mangroves for biodiversity conservation and carbon storage. Nat. Commun..

[51] Wang H., Gilbert J.A., Zhu Y., Yang X. (2018). Salinity is a key factor driving the nitrogen cycling in the mangrove sediment. Sci. Total Environ..

[52] Chambers L.G., Davis S.E., Troxler T., Boyer J.N., Downey-Wall A., Scinto L.J. (2014). Biogeochemical effects of simulated sea level rise on carbon loss in an Everglades mangrove peat soil. Hydrobiologia.

[53] Rao R.G., Woitchik A.F., Goeyens L., Van Riet A., Kazungu J., Dehairs F. (1994). Carbon, nitrogen contents and stable carbon isotope abundance in mangrove leaves from an east African coastal lagoon (Kenya). Aquat. Bot..

[54] Hossain M., Siddique M.R.H., Saha S., Abdullah S.M. (2015). Allometric models for biomass, nutrients and carbon stock in *Excoecaria agallocha* of the Sundarbans, Bangladesh. Wetl. Ecol. Manag..

[55] Mace G.M., Norris K., Fitter A.H. (2012). Biodiversity and ecosystem services: A multilayered relationship. Trends Ecol. Evol..

[56] Cai W., Yang K., Wu L., Huang G., Santoso A., Ng B., Yamagata T. (2021). Opposite response of strong and moderate positive Indian Ocean Dipole to global warming. Nat. Clim. Chang..

[57] Roose S., Ajayamohan R.S., Ray P., Mohan P.R., Mohanakumar K. (2022). ENSO influence on Bay of Bengal cyclogenesis confined to low latitudes. NPJ Clim. Atmos.

[58] Zhang H., He H., Zhang W.Z., Tian D. (2021). Upper Ocean response to tropical cyclones: A review. Geosci. Lett..

[59] Seriño M.N., Ureta J.C., Baldesco J., Galvez K.J., Predo C., Seriño E.K. (2017). Valuing the Protection Service Provided by Mangroves in Typhoon-Hit Areas in the Philippines.

[60] Singh P., Bengtsson L. (2005). Impact of warmer climate on melt and evaporation for the rainfed, snowfed and glacierfed basins in the Himalayan region. J. Hydrol..

[61] Carter H.N., Schmidt S.W., Hirons A.C. (2015). An international assessment of mangrove management: Incorporation in integrated coastal zone management. Diversity.

[62] Ahmed S., Sarker S.K., Friess D.A., Kamruzzaman M., Jacobs M., Islam M.A., Pretzsch H. (2022). Salinity reduces site quality and mangrove forest functions. From monitoring to understanding. Sci. Total Environ..

[63] Menéndez P., Losada I.J., Torres-Ortega S., Narayan S., Beck M.W. (2020). The global flood protection benefits of mangroves. Sci. Rep..

[64] Aziz A., Paul A.R. (2015). Bangladesh Sundarbans: Present status of the environment and biota. Diversity.

[65] Bhattacharyya S., Raha A.K., Mitra A. (2018). Ecotourism revenue in Sunderban Tiger Reserve. Techno Int. J. Health Eng. Manag. Sci..

[66] Segaran T.C., Azra M.N., Lananan F., Burlakovs J., Vincevica-Gaile Z., Rudovica V., Satyanarayana B. (2023). Mapping the Link between Climate Change and Mangrove Forest: A Global Overview of the Literature. Forests.

[67] Friess D.A., Adame M.F., Adams J.B., Lovelock C.E. (2022). Mangrove forests under climate change in a 2 C world. Wiley Interdiscip. Rev. Clim. Chang..

[68] Chowdhury A., Naz A., Maiti S.K. (2023). Variations in soil blue carbon sequestration between natural mangrove metapopulations and a mixed mangrove plantation: A case study from the world’s largest contiguous mangrove forest. Life.

[69] Bosire J.O., Dahdouh-Guebas F., Kairo J.G., Koedam N. (2003). Colonization of non-planted mangrove species into restored mangrove stands in Gazi Bay, Kenya. Aquat. Bot..

[70] Van Der Stocken T., Wee A., De Ryck D.J.R., Vanschoenwinkel B., Friess D.A., Dahdouh-Guebas F., Simard M., Koedam N., Webb E.L. (2019). A general framework for propagule dispersal in mangroves. Biol. Rev..

